# Tracking Extended-Spectrum β-Lactamase-Producing *Escherichia coli* Across Human Communities and Dairy Ecosystems: A One Health Investigation

**DOI:** 10.3390/antibiotics15060588

**Published:** 2026-06-09

**Authors:** Cassandra Klaas, Shawn Hoogstra, David Mahoney, Mark Lubberts, Emil Jurga, Gabriel Wajnberg, Daniella Rizzo, Richard J. Reid-Smith, Catherine Carrillo, Rhiannon L. Wallace

**Affiliations:** 1 Agassiz Research and Development Center, Agriculture and Agri-Food Canada, Agassiz, BC V0M 1A0, Canada; 2 Department of Community Health and Epidemiology, Faculty of Medicine, Dalhousie University, Halifax, NS B3H 4R2, Canada; 3 Faculty of Computer Science, Dalhousie University, Halifax, NS B3H 4R2, Canada; 4 Summerland Research and Development Center, Agriculture and Agri-Food Canada, Summerland, BC V0H 1Z0, Canada; 5 National Microbiology Laboratory, Public Health Agency of Canada, Ottawa, ON K1A 0K9, Canada; 6 National Centre for Animal Disease, Canadian Food Inspection Agency, Lethbridge, AB T1J 5R7, Canada; 7 Centre for Foodborne, Environment and Zoonotic Infectious Diseases, Public Health Agency of Canada, Guelph, ON N1G 3W4, Canada; 8 Ottawa Laboratory (Carling), Canadian Food Inspection Agency, Ottawa, ON K1A 0C6, Canada

**Keywords:** antimicrobial resistance (AMR), *Escherichia coli*, extended-spectrum beta-lactamase (ESBL), Sequence Type 648 (ST648), ST88, whole genome sequencing (WGS)

## Abstract

Background: The rising prevalence of Extended-spectrum β-lactamase-producing (ESBL) *Escherichia coli* poses a significant threat to human and animal health. Methods: To address this, we conducted a longitudinal two-year One Health study to assess ESBL *E. coli* occurrence and distribution across dairy farms, surrounding environments, and urban wastewater in a peri-urban region of Western Canada. Results: A total of 546 presumptive ESBL *E. coli* were recovered, with the highest occurrence in wastewater influent (75.9%) and calf feces (73.6%), and lowest in soil (6.3%) and surface water (18.8%). Seasonal analysis showed a significantly higher occurrence in summer compared to spring. The *bla_CTX-M-15_* gene predominated (79%), followed by *bla_TEM_* (28%) and *bla_SHV_* (9%), with most isolates harboring multiple ESBL genes. Whole-genome sequencing of 387 isolates identified 75 resistance determinants spanning nine antimicrobial classes, including 24 β-lactamase genes and 10 CTX-M variants. Ninety-four sequence types (STs), including nine novel STs, were detected. The most common STs were ST648, ST69, and ST10, with distinct distributions across sources. Plasmid analysis revealed extensive diversity, with approximately half of the plasmid types shared across multiple sample types, indicating potential horizontal gene transfer. Over 200 virulence factors were identified, including toxin genes and Shiga toxin-associated genes, primarily in calf and surface water isolates. Phylogroups A and B1 dominated samples from dairy farms, and phylogroup B2 was restricted to wastewater and surface water. Conclusions: These findings identify the environment as a reservoir for ESBL *E. coli* and reveal the unexpected predominance of the emerging MDR ST648 lineage, rather than ST131, and reinforce the need for comprehensive integrated One Health surveillance.

## 1. Background

Antimicrobial resistance (AMR) poses a serious threat to global health, jeopardizing the efficacy of drugs to treat serious infections and rolling back decades of medical advances [1]. The World Health Organization (WHO) has listed AMR as one of the top ten global health concerns [2]. AMR is a naturally occurring phenomenon; however, the overuse and misuse of critically important antimicrobials have exacerbated this problem. Drug-resistant bacteria are found in humans, animals, crops, and food, and there are increasing concerns that antimicrobials used to treat illness in humans and animals can make their way into the environment, further accelerating the development of AMR in water and soil [3]. The environment is being increasingly studied due to its role as a source and a route for the dissemination of AMR [4].

*Escherichia coli* is a versatile bacterium that inhabits the gastrointestinal tract of humans and animals and is widespread across environments. Many strains are recognized as harmless commensals [5], but all strains have the potential to harbor antimicrobial resistance genes (ARGs) [6], and some lineages possess virulence factors that enable them to cause various diseases [5]. In addition to being used as an indicator of fecal contamination, *E. coli* is increasingly used as a sentinel organism for monitoring AMR in the environment. Among the most concerning resistance phenotypes, Extended-spectrum β-lactamase producing (ESBL) *E. coli*, are of particular concern to human health as they are resistant to most β-lactam antimicrobials, including 3rd generation cephalosporins, requiring treatment with last resort drugs [7]. These bacteria are also frequently multidrug-resistant (MDR), and not surprisingly, the WHO has listed ESBL *E. coli* as a priority pathogen for new antibiotic development [8]. Resistance in ESBL *E. coli* is often encoded by genes such as *bla*_CTX-M_, *bla*_TEM_, and *bla*_SHV_, often located on plasmids that can be easily transferred between and within bacterial species [9]. The predominant ESBL genes globally are the *bla_CTX-M_* family, with more than 200 known variants [10]. Most research on ESBL-producing bacteria is focused on clinical settings [11], wastewater [12] and to a lesser extent, farm environments. Very few studies on ESBL *E. coli* investigate all three components of the One Health continuum simultaneously.

In Canada, the incidence of ESBL *E. coli* infections is increasing, with higher proportions of ESBL *E. coli* recovered from certain regions of Canada, including British Columbia [13]. In Canada, over 80% of antimicrobials are sold for use in production animals, compared to about 17% in humans [14]. However, antimicrobial use varies greatly by animal sector. The Fraser Valley is a major dairy farming region in Western Canada, producing over 75% of British Columbia’s total milk supply [15]. The peri-urban nature of this region, with dairy farms in close proximity to urban communities, may present new avenues for pathogen and AMR transmission. Both manure and waste milk from cows receiving antibiotic treatment may contain antibiotic residues and resistant bacterial strains that could accumulate and spread through the environment [16]. Since antimicrobial-resistant bacteria can be transmitted from animals to humans through shared environments, a One-Health approach recognizing the interconnectedness of human, animal, and environmental health is necessary to identify areas for AMR mitigation [17]. Without mitigation, there is a risk that antimicrobial use in dairy operations could further propagate AMR [18]. Despite this, little is known about the occurrence of ESBL bacteria across the One-Health continuum of this peri-urban region in Western Canada. This study investigated the occurrence of ESBL *E. coli* on commercial dairy farms, surrounding environments, and urban wastewater within the Fraser Valley. We used whole genome sequencing (WGS) to compare the diversity of ESBL *E. coli* and analyze the distribution of ARGs and mutations, metal resistance genes, virulence factors, and plasmids through a two-year longitudinal One-Health study.

## 2. Results

### 2.1. Occurrence of ESBL E. coli Across the One Health Continuum

Over a two-year period, 546 presumptive ESBL *E. coli* were isolated on CHROMagar ESBL^TM^ media. Occurrence of presumptive ESBL-producing isolates was highest in wastewater influent and calf fecal samples, occurring in 75.93% (41/54) of collected influent samples, and 73.61% (106/144) of collected calf samples (Table 1). Occurrence was lowest in environmental samples, occurring in 6.25% (6/96) of soil samples, and 18.75% (27/144) of surface water samples. The other sample types included milking cow feces, dry cow feces, manure pit, and wastewater effluent, which had ESBL *E. coli* detected in 33.33–47.92% of the samples. Of note, calf feces and wastewater influent had a significantly higher occurrence of ESBL-producing *E. coli* compared to the other sample sources (*p* < 0.01, Fisher’s Exact Test), and manured soil and surface water samples had a significantly lower occurrence compared to farm samples (*p* < 0.0009, Fisher’s Exact Test). No significant differences were found between dry cow feces, milking cow feces, and the manure pit (*p* > 0.05, Fisher’s exact test).

Occurrence of ESBL E. coli was significantly higher in summer (50.44%; 115/228 samples) than in spring (35.53%; 81/228 samples; *p* = 0.0078, Fisher’s Exact Test, Bonferroni adjustment), with no significant differences found between any of the other seasons. When comparing seasonal differences within sample types, only a significant difference was found between spring and summer samples for milking cows (*p* = 0.0194, Fisher’s Exact Test, Bonferroni adjustment), with no significant seasonal variations detected in any of the other sample types.

### 2.2. Distribution of ESBL Genes Among E. coli Isolates

**Figure 1:**
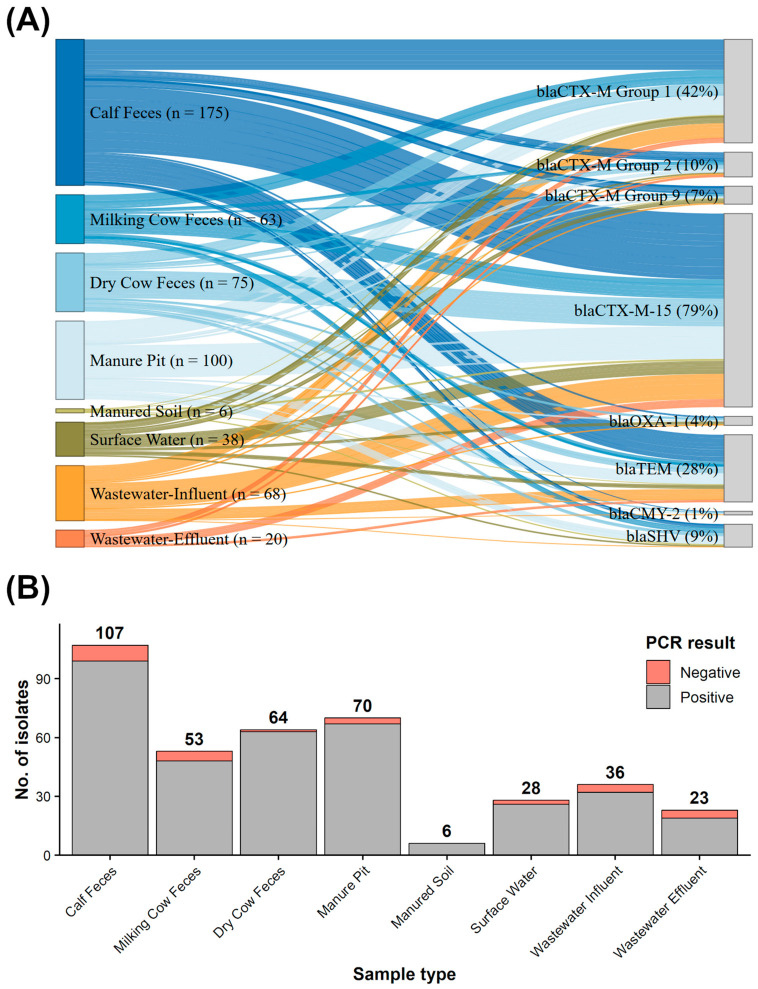
(**A**) Sankey plot displaying the association between sample type and ESBL gene among the 513 *E. coli* isolates positive for an ESBL gene. Percentages in parentheses indicate the proportion of *E. coli* recovered on CHROMagar^TM^ ESBL (*n* = 545) positive for each gene by PCR. (**B**) Summary of *E. coli* isolates (*n* = 387) selected for whole genome sequencing, including the 27 isolates that were ESBL gene-negative based on PCR.

Of the ESBL genes screened via PCR, *bla*_CTX-M-15_ was the most common, occurring in 79% (432/545) of isolates. The *bla*_TEM_ gene was detected in 28% (150/545) of isolates, and *bla*_SHV_ was detected in 9% (51/545) of isolates (Figure 1A). The *bla*_OXA-1_ and *bla*_CMY-2_ genes were detected the least, with only 4% (20/545) of isolates testing positive for *bla*_OXA-1_, and 1% (8/545) testing positive for *bla*_CMY-2_. All ESBL genes were detected in bovine, environmental, and wastewater samples, and no genes were exclusive to a specific sample type (Figure 1A). Approximately one-third of ESBL *E. coli* isolates (167/545) possessed only a single ESBL gene, while the vast majority (63.6%; 347/545) of isolates screened via PCR had two or more of the target genes (Table S1). A small number (5.9%; 32/545) of isolates were negative for all ESBL genes tested by PCR. Isolates with no ESBL genes detected were found across all sample types except for soil. A subset of these isolates, seemingly lacking ESBL genes, was selected for WGS (Figure 1B).

### 2.3. Antimicrobial Resistance Profile

**Figure 2:**
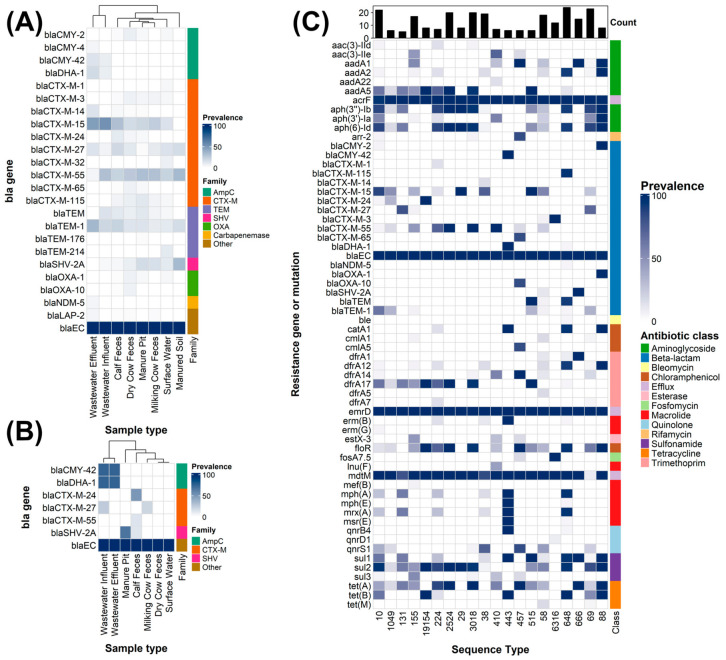
(**A**) Heatmap showing the prevalence (%) of β-lactamase resistance genes across isolates from each sample type. (**B**) Heatmap summarizing β-lactamase resistance genes detected in the 27 *E. coli* isolates that tested negative for ESBL genes by PCR. (**C**) Heatmap summarizing the prevalence of all resistance genes and mutations found in the 20 most common Sequence Types. Counts on the top indicate the number of isolates belonging to each ST.

Genome analysis identified 75 different resistance genes or mutations, including 24 *bla* genes, amongst our collection of 387 *E. coli* isolates. All isolates possessed at least one β-lactamase gene, with *bla_EC_* (100.0%; 387/387), followed by *bla_CTX-M-15_* (27.1%; 105/387), *bla_CTX-M-55_* (25.3%; 98/387), and *bla_TEM-1_* (16.5%; 64/387) being the most prevalent (Figure 2A). Isolates from urban wastewater clustered together and separated from the bovine and environmental isolates, highlighting their shared β-lactam resistome signatures (Figure 2A). Among the 27 isolates that initially screened negative for ESBL genes, all carried at least one *bla* gene (Figure 2B), although 40.7% (11/27) only possessed *bla_EC_*. Genes associated with multidrug efflux pumps (*acrF*, *emrD*, and *mdtM*) were present in >90% of our isolates (Table S1). Resistance genes associated with nine different classes of antimicrobials were detected. Among the other genes not associated with β-lactams or efflux pumps, *sul2* (56.3%; 218/387), followed by *tet(A)* (50.1%; 194/387), *aph(3″)-Ib* (49.4%; 191/387), and *aph(6)-ld* (49.1%; 190/387) were the most common. Ten of the resistance determinants were only detected once and included 4 β-lactam genes: *bla_CMY-4_*, *bla_CTX-M-1_*, *bla_NDM-5_*, and *bla_TEM-176_* (Table S1).

**Figure 3:**
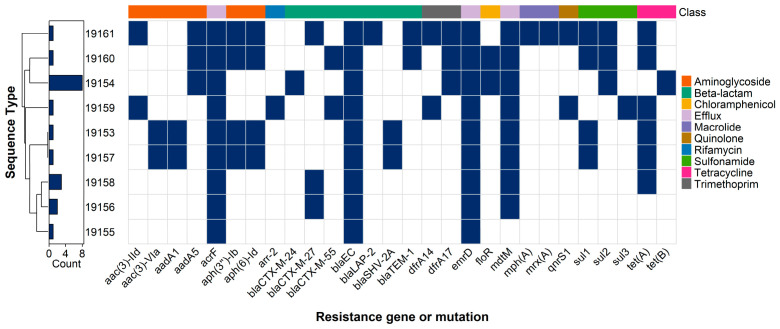
Heatmap showing the prevalence of resistance genes and mutations found in *E. coli* isolates (*n* = 19) representing 9 novel Sequence Types (STs). Counts on the left indicate the number of isolates belonging to each ST.

These isolates represented 94 different sequence types (STs), which included nine novel STs reported for the first time here (Table S1 and Figure 3). The most common were ST648 (24/387), ST69 (23/387), and ST10 (22/387), with 46 singleton STs (Table S1). Among the 20 most common STs, which represented 257 isolates, diverse resistance profiles were observed (Figure 2C). ST10, one of the top three STs, appeared in all sample types except surface water, while no ST was present across all sample types. Among the top 20 STs, ST515 (*n* = 6) was only found in calf feces, and ST443 (*n* = 6) was only found in wastewater influent.

The most common STs varied by sample type (Table 2), with dominant resistance profiles containing 6–12 genes and conferring resistance to 2–6 antimicrobial classes.

Nineteen *E. coli* belonged to 9 novel STs identified for the first time in this study (Figure 3). These isolates harbored between 3 and 19 different resistance genes or mutations, including 7 different *bla* genes. In addition to the *bla_EC_* gene, each of the novel isolates possessed 1–3 additional *bla* genes (Figure 3). Of note, one isolate (AMC1039) recovered from wastewater effluent belonging to ST19161 had 19 resistance determinants spanning 7 classes of antimicrobials (Table S1 and Figure 3).

### 2.4. Metal, Biocide, and Heat Resistance

**Figure 4:**
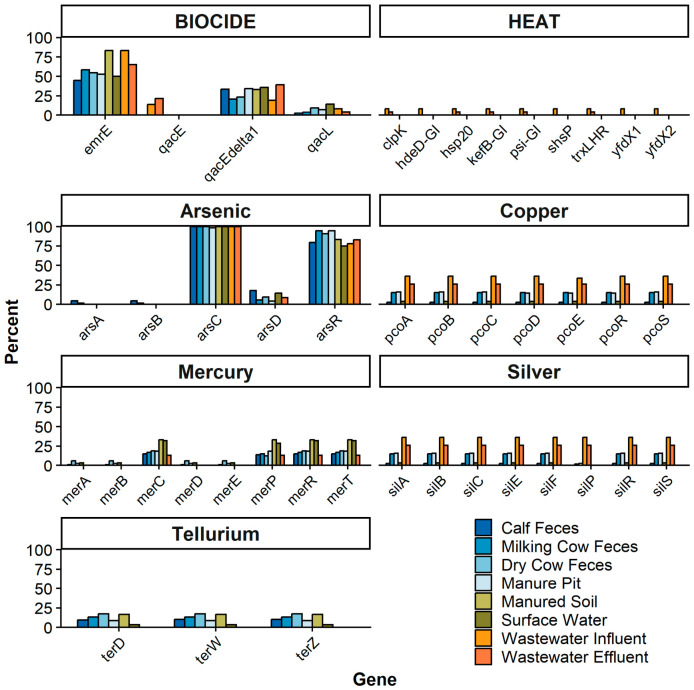
Bar graphs illustrating the percent of ESBL *E. coli* isolates (*n* = 387) carrying biocide or heat tolerance genes and metal resistance genes by sample type.

Among our collection of 387 ESBL *E. coli* isolates, we found 4 and 9 genes associated with biocide resistance and heat tolerance, respectively (Figure 4). The *emrE* multidrug efflux transporter was the most common biocide gene present in 45–83% of isolates from each sample type. Some stress genes, namely *qacE* and all 9 heat tolerance genes, were exclusive to urban wastewater isolates, although these genes were uncommon (present in <8 isolates). We also identified 31 metal resistance genes in our collection of isolates (Figure 4). The *arsC*, followed by *arsR*-associated arsenic, were the most common and were found in 75–100% of isolates from each sample type, with the occurrence of the other metal genes ranging from zero to 36%. Genes associated with copper and silver were noticeably more prevalent in isolates from wastewater influent and effluent. A subset of mercury genes (*merA*, *merB*, *merD*, and *merE*) and all three tellurium genes (*terD*, *terW*, and *terZ*) were detected in all sample types except for urban wastewater.

### 2.5. Mobile Genetic Elements

**Figure 5:**
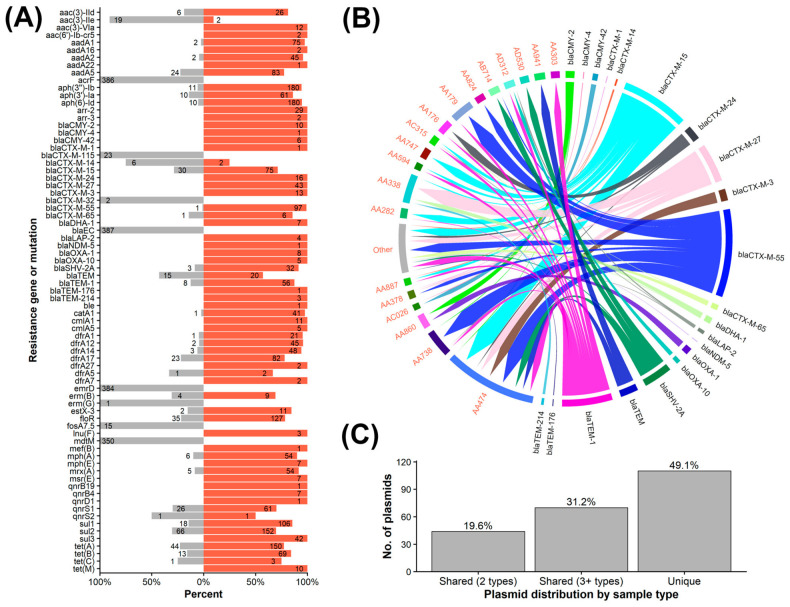
(**A**) Mirror plot showing the distribution of resistance genes and mutations as chromosomal (gray) or plasmid-borne (red). The gene counts are shown at the end of each bar. (**B**) Circos plot representing associations between *bla* (Extended-spectrum β-lactamase) antibiotic resistance genes (black text) and their host plasmids (red text). Plasmid types occurring <5 times are grouped as “Other”. (**C**) Bar plot summarizing the distribution of 224 plasmid types as either unique to a single sample type or shared across multiple sample types.

Among the 75 different resistance genes and mutations found, 8 were chromosomal, and 35 were plasmid-borne, with the remainder associated with chromosomal and plasmid DNA (Figure 5A). Three β-lactamase genes, *bla_EC_*, *bla_CTX-M-32_*, and *bla_CTX-M-115_*, were not associated with plasmids, while the remaining 21 *bla* genes were associated with 45 different plasmids (Figure 5B). The most common plasmids harboring *bla* genes were AA474, AA338, AA738, and AA179. The most commonly detected ESBL genes, *bla_CTX-M-55_*, *bla_CTX-M-15_*, and *bla_TEM-1,_* were each associated with over 7 different plasmid types (Figure 5B). Among the 224 plasmid types identified amongst our collection of 387 *E. coli* isolates, approximately half (50.8%) were found in isolates from two or more different sample types, while 49.1% of the plasmids were found in only a single sample type (Figure 5C).

### 2.6. Virulence Factors

In total, we found 230 virulence factors amongst our 387 ESBL *E. coli* isolates, with 27 of these genes present in >90% of the isolates. The total number of virulence factors per isolate did not differ significantly by sample type. Over half of the virulence factors (137/230) represented singleton genes that were only found in one isolate, with the greatest number of singleton virulence factors found in isolates from milking cow (28 virulence factors) and manure pit samples (25 virulence factors) (Table 3). Among the 230 virulence factors, 13 were associated with toxin genes, with *astA* (33/387), followed by *hlyA* (16/387), and *senB* (14/387) being the most common. Only two toxin genes were found in isolates from surface water and wastewater effluent, while 11 toxin genes were found in isolates from calf feces (Table 3). Shiga-toxin genes were found in nine *E. coli* isolates from calf feces and one from surface water. Six Shiga toxin-producing *E. coli* (STEC) isolates from calf feces belonged to ST515 and carried genes encoding the *Stx2b* and *Stx2d* subtypes. The remaining fecal isolates were identified as ST342 (*Stx1a*), ST446 (*Stx2d*), and ST937 (*Stx1a*, *Stx2b*). Notably, the ST342 isolate was the only one to carry the *eae* gene, identifying it as EHEC. Additionally, a single STEC isolate from surface water was identified as ST101 and carried genes for the *Stx1a* subtype.

### 2.7. Phylogenetic Analysis

**Figure 6:**
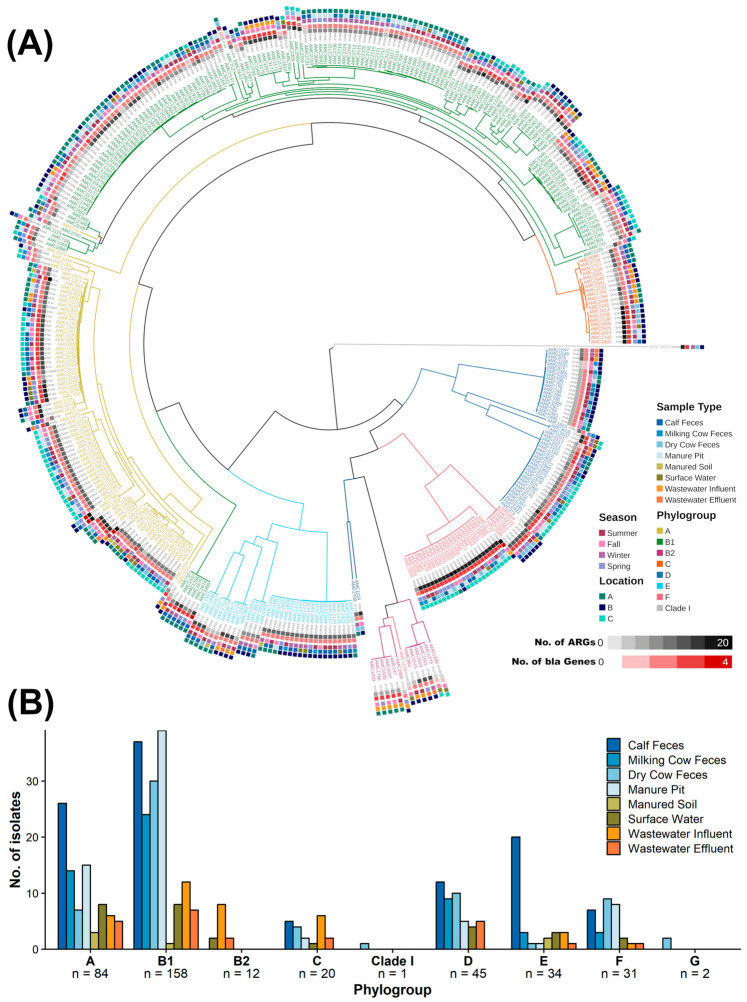
(**A**) Maximum likelihood phylogenetic tree of ESBL *E. coli* recovered in this study (*n* = 387). (**B**) Bar plot showing the distribution of isolates in each phylogroup by sample type.

A Maximum-Likelihood core genome phylogeny was built to ascertain the diversity of the 387 ESBL *E. coli* isolates recovered from six commercial dairy farms and two wastewater treatment facilities in the Fraser Valley region of British Columbia. The core genome-based phylogenomic tree for *E. coli* showed clustering by phylogroup, ST, and geographic location (Figure 6A). Isolates from urban wastewater (e.g., ST73, ST219, ST410, ST443) were generally more closely related to other isolates from wastewater than bovine or environmental isolates. Pairwise SNP differences between isolates ranged from zero to 122,896 SNPs (Table S2). The single isolate (AMC2859) belonging to Clade I was not closely related to any other isolates obtained in this study (46,361–122,896 SNPs). Eight isolates recovered from bovine feces and one from surface water belonging to ST88 recovered from a single conventional dairy farm harbored 18 resistance genes, including three *bla* genes (*bla_CMY-_2*, *bla_EC_*, and *bla_OXA-1_*) (Figure 6A and Table S1). Twenty-four isolates belonged to ST648 and possessed 17–19 ARGs, with the exception of one isolate (AMC1898) that had only 8 ARGs. Among the ST648 isolates, which were recovered from all sample types except soil and wastewater effluent, each possessed 2–3 *bla* genes with the exception of AMC1173, obtained from wastewater influent that possessed 4 *bla* genes (*bla_DHA-1_*, *bla_EC_*, *bla_NDM-5_*, and *bla_TEM-1_*) (Figure 6A and Table S1).

Phylogroups B1 (158/387) were most prevalent, followed by phylogroup A (84/387), with isolates from all sample types distributed across most phylogroups (Figure 6B). Isolates belonging to phylogroup B2 were only recovered from wastewater (*n* = 10) and surface water (*n* = 2). Only one isolate belonging to Clade I and two belonging to phylogroup G, both from dry cow feces, were found.

**Figure 7:**
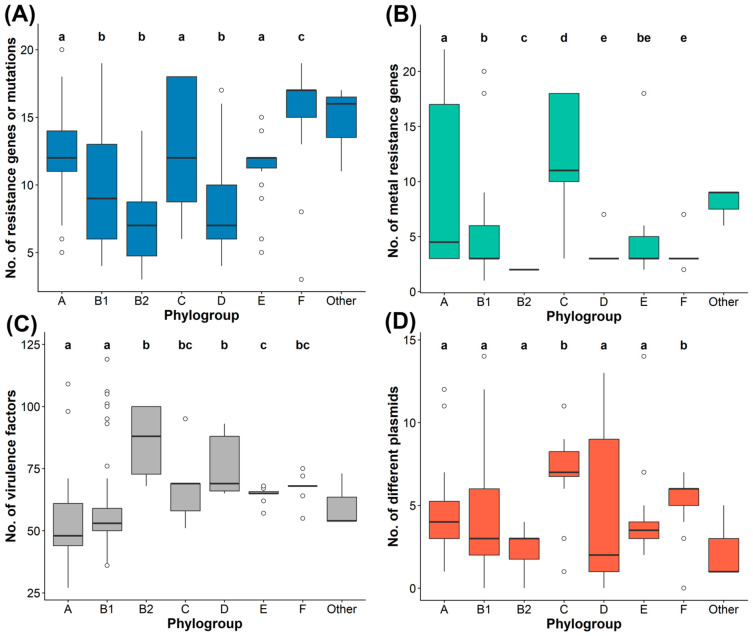
Boxplot illustrating the total number of (**A**) antimicrobial resistance genes and mutations, (**B**) metal resistance genes, (**C**) virulence factors, and (**D**) plasmids per *E. coli* isolate (*n* = 387), stratified by phylogroup. Phylogroups represented by <5 isolates were grouped as “Other”. Boxes represent the interquartile range (Q1–Q3), the horizontal line within each box indicates the median, whiskers show the range of non-outlier values, and points denote outliers. Different letters above the whiskers indicated statistically significant differences (*p* < 0.05).

Phylogroup F isolates carried significantly (*p* < 0.05) more resistance genes than the other phylogroups, whereas phylogroup C isolates harbored more metal resistance genes (Figure 7A,B). Phylogroup A and B1 isolates carried significantly (*p* < 0.05) fewer virulence factors (Figure 7C), while isolates belonging to phylogroups C and F carried significantly (*p* < 0.05) more plasmids per isolate (Figure 7C).

## 3. Discussion

ESBL *E. coli* are an increasing public health threat in Canada and globally. The findings of this study, demonstrating the presence of ESBL *E. coli* across interconnected systems (bovine, environment, and human wastewater), highlight the multifaceted ecology of AMR and the role of multiple reservoirs in its persistence and transmission. The substantial genetic diversity observed in resistance determinants and plasmids indicates that resistance is not confined to a specific lineage or ST; rather, mobile genetic elements are widely shared across sources.

The occurrence of ESBL *E. coli* varied significantly across sample type reservoirs, with wastewater influent and calf feces showing the highest levels of ESBL *E. coli* across all sample types. The high prevalence of ESBL bacteria in calves is consistent with previous studies identifying youngstock as important reservoirs and amplifiers of ESBL *E. coli,* with reported prevalences exceeding 70% in some production systems [9,18].

This is likely explained by the fact that calves, with their developing immune systems, are often targeted for antimicrobial treatment for certain diseases (e.g., respiratory illness and diarrhea), while adult dairy cows are treated less often and more selectively [19]. Calves are often also fed waste milk produced by cows receiving antibiotics, which may contain antibiotic residues, therefore adding an additional selective pressure to this age group. The elevated occurrence in wastewater influent reinforces its role as a convergence point for resistant bacteria originating from human sources. Our findings are in agreement with Maric et al., who found ESBL *E. coli* in 93% of wastewater influents in Slovenia [20]. A recent systematic review and meta-analysis estimated the global prevalence of ESBL *E. coli* in wastewater at 24.81%, with higher levels reported in America (39.91%) than in Europe, and an overall increasing trend worldwide [21]. In this study, the occurrence of ESBL *E. coli* was significantly lower in the effluent (33.33%) than in the influent, suggesting that the wastewater treatment processes reduce the load of these organisms and may limit their release into the environment. In contrast, the significantly lower detection of ESBL-producing *E. coli* in soil and surface water supports the notion that environmental matrices may act as secondary reservoirs, where dilution, environmental stressors, and reduced host association limit bacterial persistence. This pattern aligns with previous work demonstrating lower recovery rates of ESBL-producing organisms in environmental samples relative to fecal and wastewater sources [20]. Milking cow feces had a ~10% lower prevalence of ESBL *E. coli* compared to dry cow feces and manure pit samples, likely reflecting sampling from untreated animals and reduced antimicrobial selection pressure within the milking herd. The detection of ESBL *E. coli* in over one-third of milking cow samples indicates that these organisms can persist even in the absence of direct antimicrobial exposure, maintaining the potential for resistance gene transmission. The intermediate prevalence observed in dry cows was expected, as many dairy producers in this region practice blanket dry cow therapy, treating all non-lactating cows with an antimicrobial such as cephapirin benzathine (first-generation cephalosporin antibiotic) to treat and prevent infections such as mastitis. Overall, these findings highlight the uneven distribution of ESBL *E. coli* across different cow production stages and interconnected sources.

Seasonal variation provides additional insight into the ecological dynamics of ESBL *E. coli*, with a significantly higher occurrence in summer compared to spring. This finding is consistent with studies reporting increased prevalence of AMR bacteria during warmer months [22,23], potentially driven by enhanced bacterial growth, survival, and transmission under elevated temperatures, as well as seasonal changes in farm management practices. However, the limited seasonal differences observed within individual sample types—apart from milking cow feces—suggest that these effects may be context-specific and influenced by localized factors. Collectively, these findings underscore the importance of considering both reservoir-specific and temporal dynamics in understanding the persistence and dissemination of ESBL *E. coli*, and reinforce the need for targeted, One Health-based surveillance and mitigation strategies.

Our preliminary PCR screening of *E. coli* isolates to confirm the ESBL genotype showed that *bla_CTX-M-15_* was the most common ESBL gene, consistent with global reports identifying CTX-M-type enzymes as the dominant ESBL genotype in *E. coli* from human, animal, and environmental sources [10,12]. In contrast, *bla_TEM_* (28%) and *bla_SHV_* (9%) were less frequently detected, reflecting the well-documented shift from TEM- and SHV-type ESBLs toward CTX-M variants [24]. The low prevalence of *bla_OXA-1_* (4%) and *bla_CMY-2_* (1%) is also in line with previous studies [7,12,25], where these genes are typically detected at lower frequencies. Importantly, all ESBL genes were identified across bovine, environmental, and wastewater samples, with no gene restricted to a specific source, supporting the widespread dissemination of resistance determinants across interconnected reservoirs. The majority of isolates carried multiple ESBL genes (63.6%), a pattern consistent with other studies reporting co-occurrence of resistance genes on mobile genetic elements such as plasmids [12]. Finally, a small proportion of isolates (5.9%) lacked detectable ESBL genes by PCR despite growing on CHROMagar ESBLTM media, suggesting the presence of less common or uncharacterized β-lactamase genes, and highlighting the value of complementary approaches such as WGS for comprehensive resistance profiling. Through WGS, we identified 75 ARGs, 24 *bla* genes, including 10 *bla_CTX-M_* variants. Carbapenemase-encoding genes were generally not detected, with *bla_NDM-5_* found in a single wastewater isolate, and other genes, such as *bla_OXA-48_* and *bla_KPC_*, were not found. The *bla_EC_* gene, encoding the chromosomal AmpC, associated with intrinsic β-lactam resistance [26], was present in all of our isolates. Of note, 11 isolates carried only the intrinsic *bla_EC_* gene without additional *bla* genes detected by PCR. Because phenotypic ESBL confirmation was not performed, these isolates cannot be confirmed as ESBL producers and may represent isolates with alternative β-lactam resistance mechanisms, resistance determinants not included in our molecular screening panel, or false-positive growth on CHROMagar ESBL.

Multi-locus sequence typing of 387 *E. coli* isolates revealed substantial genomic diversity, comprising 94 STs, which is consistent with previous One Health investigations of ESBL *E. coli*, which report genetically diverse populations spanning numerous lineages [27,28]. Our collection of isolates was dominated by ST648, followed by ST69 and ST10. ST648 isolates were recovered from bovine (calf and cow feces), environmental (surface water), and urban wastewater (influent), and were more frequently detected than the globally disseminated ST131 (*n* = 5). ST648 isolates generally possessed 20 ARGs representing over 6 drug classes, including 3–4 β-lactamase genes. ESBL *E. coli* ST648 is an emerging, globally distributed, MDR, and highly virulent lineage comparable to ST131 with major implications for public health, infection control, and antimicrobial stewardship [29]. In agreement with other reports, our findings suggest ST648 is a generalist with the capability of frequent cross-species transmission, thriving in various environments, similar to ST131 [30]. We also recovered 8 ESBL *E. coli* isolates from bovine (calf and cow feces) and environmental (surface water) samples obtained from a single dairy farm in three different seasons, belonging to ST88, an emerging MDR lineage that has been less well described [12,31]. Each ST88 isolate harbored *bla_CMY-2_*, *bla_EC_*, and *bla_OXA-1_*, as well as 15 other resistance genes associated with at least 5 different drug classes. In comparison to ST648 isolates, *E. coli* belonging to ST88 tend to be associated with animals and environmental samples [28], which was the case in this study. Despite the dominance of a few lineages, overall strain diversity remained high, with 46 singleton STs and extensive variation in STs across sample sources. No single ST was shared across all sample types, although ST10 showed the broadest distribution, occurring in all matrices except surface water. The identification of nine novel STs further highlights ongoing diversification within these interconnected reservoirs, including ST19161, which carried 19 ARGs spanning more than 6 drug classes. We also found several isolates belonging to pandemic pathogenic STs such as ST38, ST58, ST73, ST127, and ST410 [31]. Overall, ST131—often considered the dominant extraintestinal pathogenic *E. coli* lineage globally—was relatively rare in this study, while other pathogenic STs were more prominent in this agro-ecosystem.

The distribution of biocide, heat-tolerance, and metal-resistance genes in our ESBL *E. coli* collection reflects the selective pressures characteristic of different environments. The disinfectant resistance gene *qacE* gene and all heat-tolerance genes were detected exclusively in urban wastewater isolates; although uncommon, these findings are in agreement with other studies that have shown wastewater to be a reservoir for stress-adaptation genes in *E. coli* [32]. Our findings are consistent with reports that wastewater microbiomes harbor diverse, low-frequency resistance and stress genes [33]. Arsenic-associated genes such as *arsC* and *arsR* were highly prevalent, whereas *arsB*—which encodes the essential arsenite efflux pump—was detected in fewer than 5% of isolates, suggesting that most isolates lacking *arsB* are likely sensitive to arsenic [34]. Genes associated with copper and silver resistance were enriched in wastewater influent and effluent, likely reflecting exposure to metal-based antimicrobials and industrial inputs, a pattern also observed in other wastewater surveillance studies [34,35]. Interestingly, tellurium-resistance genes (*terD*, *terW*, and *terZ*) were absent from urban wastewater but present in all other sample types, suggesting that these determinants may be more characteristic of agricultural or environmental reservoirs than municipal systems. In contrast, urban wastewater may act as a reservoir for stress-adaptation traits, including metal resistance genes (copper and silver) that are less common in agricultural or natural environments.

The distribution of resistance determinants in our isolates highlights the dominant role of plasmids in ESBL gene mobility. Nearly half of the 75 resistance genes or mutations identified were plasmid-borne, and 21 of the 24 *bla* genes were linked to 45 different plasmid types, demonstrating extensive plasmid-mediated exchange. The most common ESBL genes—*bla_CTX-M-55_*, *bla_CTX-M-15_*, and *bla_TEM-1_*—were each associated with more than seven plasmid backbones, consistent with their known ability to spread across diverse plasmid lineages [36,37]. Several plasmids (AA474, AA338, AA738, AA179) appeared repeatedly across isolates, suggesting they may act as successful vehicles for ESBL dissemination. The presence of 224 plasmid types across 387 isolates, with roughly half occurring in multiple sample types, indicates substantial plasmid flow between wastewater, agricultural, and environmental compartments, while the remaining plasmids restricted to a single source likely represent niche-adapted or recently acquired elements [38]. A key limitation of our study is that plasmid structures were inferred from Illumina short-read sequencing, which cannot fully resolve plasmid boundaries or distinguish co-located resistance genes with complete certainty. Overall, these patterns show that plasmid diversity and mobility are key drivers of ESBL spread in our study system.

The virulence factors found in our collection of ESBL *E. coli* were highly diverse, with 230 distinct virulence factors identified, but only 27 were present in more than 90% of isolates, indicating a small core virulome and a large array of accessory virulence factors. Toxin-associated genes were relatively uncommon overall, yet their distribution revealed clear ecological patterns. The most frequently detected toxin genes—*astA, hlyA*, and *senB*, typically associated with uropathogenic *E. coli* [39,40], occurred predominantly in isolates from calf feces, where 11 different toxin genes were identified, compared with only two toxin genes detected in surface water and wastewater effluent. This enrichment of toxin genes in calf-associated isolates aligns with the known prevalence of enterotoxigenic and enterohemorrhagic *E. coli* in calves [41]. Shiga toxin genes were rare but similarly concentrated in calf feces, with only a single detection of a strain unrelated to the nine calf isolates in surface water, suggesting limited environmental dissemination of STEC-associated virulence on these dairy farms. Notably, more than half of all virulence factors were singletons found in only one isolate, with the highest numbers occurring in milking cow and manure pit samples. This high proportion of unique virulence genes points to substantial micro-diversity within agricultural environments and suggests that livestock-associated niches may serve as reservoirs for rare or emerging virulence traits.

The core-genome phylogeny revealed substantial genetic diversity among the 387 ESBL *E. coli* isolates, with clear clustering by phylogroup, ST, and sampling location. Wastewater isolates formed distinct clades dominated by human-associated lineages such as ST73, ST219, ST410, and ST443, reflecting the different host and ecological pressures shaping these populations. In contrast, agricultural isolates were broadly distributed, consistent with the predominance of phylogroups A and B1—lineages commonly associated with livestock and environmental reservoirs. The restriction of phylogroup B2 to *E. coli* isolates from wastewater and surface water samples highlights the human-associated nature of this group. Phylogroup B2 dominated clinical samples obtained from rural hospitals in Thailand [42] as well as wastewater in Japan [43], suggesting high survivability in water. Phylogroup F included two STs—the emerging MDR ST648 lineage and ST457—that persisted on the same farm across multiple seasons. ST457 was confined to manure pit samples, while ST648 was detected more broadly in calf feces, dry cow feces, and manure pit samples, suggesting wider circulation within the herd. The detection of a single MDR Clade I isolate belonging to ST3042, distantly related to any other isolate in our collection, underscores the overall heterogeneity of the population. Together, these patterns highlight a highly structured yet interconnected ESBL *E. coli* population, shaped by host-specific selection pressures but with evidence of persistence and potential exchange across environmental and agricultural reservoirs.

## 4. Conclusions

In summary, this study demonstrates that ESBL *E. coli* are not only widespread but also persist across bovine, environmental, and human-associated sources within a confined region of Western Canada over a two-year period. The population structure was highly diverse, yet included clinically significant and emerging MDR lineages, notably ST648, which was the predominant ST and consistently detected across nearly all sampled sources and seasons. The particularly high prevalence of ESBL *E. coli* in wastewater influent and calf feces points to both anthropogenic and agricultural systems as possible areas for intervention. Finally, the identification of environmental reservoirs further emphasizes that AMR mitigation cannot be achieved through clinical or agricultural measures alone. These findings highlight the need for a coordinated One Health approach to AMR surveillance that integrates human, animal, and environmental data.

## 5. Materials and Methods

### 5.1. Sample Collection and Bacterial Isolation

*E. coli* isolates (*n* = 1562) were previously isolated from samples collected seasonally from August 2022 to July 2024 at six commercial dairy farms (3 organic and 3 conventional), surrounding environments, and two urban wastewater treatment facilities in a One-Health surveillance study in the Fraser Valley region of British Columbia [44]. Samples were collected in triplicate and included dairy cow feces from calves, dry cows, milking cows, and manure pits, as well as environmental samples: soil and surface water collected in proximity to where the cows were housed. To capture the human component of the One Health continuum, in parallel, wastewater influent and effluent samples were collected in triplicate from wastewater treatment facilities located in the same cities as the dairy farms. Of the 1562 *E. coli* isolates recovered as part of our previous study, 546 isolates were identified as presumptive ESBL *E. coli*. Briefly, 10 g of the sample was diluted in Buffered Peptone Water (BPW, Becton, Dickinson and Company; Mississauga, ON, Canada) and plated on CHROMagar^TM^ ESBL media (Dalynn Biologicals; Calgary, AB, Canada). Plates and the inoculated BPW were incubated overnight at 37 °C. If no pink colonies indicative of *E. coli* were observed on the CHROMagar^TM^ ESBL plates, a loopful of the BPW non-selective enrichment was streaked on fresh CHROMagar^TM^ ESBL plates and incubated overnight at 37 °C to try to obtain more ESBL *E. coli* colonies. Surface water and wastewater samples were processed by filtering 100 mL at varying dilutions through a 0.45 µm membrane filter and plating onto CHROMagar^TM^ ESBL media and incubated overnight at 37 °C. Isolates that successfully grew on ESBL CHROMagar^TM^ were deemed to have an ESBL-producer phenotype and were classified as “presumptive ESBL isolates”, pending confirmation via whole-genome sequencing. Up to two isolates were purified on Levine Eosin Methylene Blue (EMB) agar (Becton, Dickinson and Company, Franklin Lakes, NJ, USA) and incubated at 37 °C for 18–20 h. Presumptive ESBL *E. coli* were grown in Tryptic Soy Broth (TSB, Becton, Dickinson and Company; Sparks, MD, USA) and preserved in 25% glycerol at −80 °C.

### 5.2. Bacterial Isolation and Identification

DNA was extracted from overnight TSB cultures, and the identity of presumptive *E. coli* isolates was confirmed using PCR targeting the *uspA* (884 bp) gene [45]. A simplex and two multiplex PCRs are described by Adator, Narvaez-Bravo, Zaheer, Cook, Tymensen, Hannon, Booker, Church, Read and McAllister [7] were modified to screen for ESBL genes to confirm *ESBL genotypes*. Primer sets were identified and developed by [46,47,48,49] (Table 4). ESBL *E. coli* DPL2034 and *Klebsiella pneumoniae* OLC4744 and OLC4850, which had previously been whole genome sequenced, were kindly provided by Dr. Catherine Carrillo (Canadian Food Inspection Agency, Ottawa Laboratory) and Dr. Dominic Poulin-Laprade (AAFC, Sherbrooke RDC) for use as internal controls in addition to isolates from the Agassiz Microbial collection (*K. pneumoniae* AMC1988 and AMC1295). The first multiplex targeted the *bla*_SHV_ (237 bp) [48], *bla*_TEM_ (445 bp) [48], *bla*_OXA-1_ (564 bp) [47], and *bla*_CMY-2_ (1000 bp) [49] genes. Each 25 uL PCR comprised 1X OneTaq Mastermix, 0.2 µM of the forward and reverse primers targeting the *bla*_SHV_, *bla*_TEM,_ and *bla*_CMY-2_ genes, 0.4 uM of the forward and reverse primers targeting the *bla*_OXA-1_ gene, 2.2 mM of MgCl_2_ (New England Biolabs, Ipswich, MA, USA), and 2 µL of DNA. Amplification was carried out as follows: initial denaturation at 95 °C for 15 min; 30 cycles of 94 °C for 60 s, 53 °C for 90 s, and 72 °C for 60 s; and a final elongation step at 72 °C for 10 min. The second multiplex targeted the *bla*_CTX-M_ group 1 (633 bp), 2 (404 bp), and 9 (561 bp) genes [47]. Each 25 µL PCR comprised 1X OneTaq Mastermix, 0.2 µM of the forward and reverse primers targeting the *bla*_CTX-M_ group 2 gene and the reverse primer targeting the *bla*_CTX-M_ group 1 gene, 0.4 µM of the forward primer targeting the *bla*_CTX-M_ group 1 gene and the forward and reverse primers targeting the *bla*_CTX-M_ group 9 gene, 4.2 mM of MgCl^2^, and 2 µL of DNA. Amplification was carried out as follows: initial denaturation at 95 °C for 15 min; 30 cycles of 94 °C for 60 s, 60 °C for 60 s, and 72 °C for 60 s; and a final elongation step at 72 °C for 10 min. Finally, each 25 µL simplex PCR targeting the *bla*_CTX-M-15_ (593 bp) gene [46,48] comprising 1X OneTaq Mastermix, 0.2 µM of the forward and reverse primers, and 2 µL of DNA. Amplification conditions were the same as those for the second multiplex targeting the same gene family. Isolates with positive amplification of any of the targeted genes above were identified as ESBL producers.

### 5.3. Genome Sequencing and Analysis

After PCR confirmation of the ESBL genotype, isolates were split into two groups: those that did not test positive for any of the ESBL genes (*n* = 33), and those that returned at least one positive result (*n* = 513). One isolate per sample was randomly selected (seed = 123) from each subset for whole genome sequencing (WGS) using R v4.5.3 [50], yielding a total of 387 selected *E. coli* isolates out of the 546 collected isolates. Genomic DNA was extracted from these 387 *E. coli* isolates using the Qiagen DNeasy Blood and Tissue Kit (Qiagen, Hilden, Germany) as described previously in our generic *E. coli* study [44]. Sequencing libraries were prepared using Illumina DNA Prep (M) Tagmentation (96 Samples, IPB) kit with Index sets A, B, C, and D, using 400 ng of DNA for each isolate. Short-read WGS was performed on an Illumina NextSeq 1000 platform using 600-cycle NextSeq^TM^ 1000/2000 P2 XLEAP-SBS^TM^ Reagent Kit. Sequencing was performed at the Canadian Food Inspection Agency in Ottawa.

Genome assembly was performed using Trimmomatic v.0.39 [51] and SPAdes v.3.15.2 [52] to filter and assemble the raw paired-end reads, respectively. Assembly statistics were assessed using QUAST v.5.0.2 [53]. Kraken2 v.2.0.9 [54] with the MiniKraken2_V1 database used to infer and verify the taxonomic identity of each isolate. Genome characterization was performed using the VMR bioinformatics pipeline (available at https://github.com/grdi-amr/vmr-bioinformatics-pipeline-nf) [accessed on 15 January 2026]. Analysis tools were used with default settings unless noted. Sequence Types (STs) and phylogroups were determined in silico using multi-locus sequence typing (Achtman 7 gene MLST Scheme) using mlst v.2.23.0 [55] and Clermont Typing [56], respectively. Novel sequence types (STs) were manually verified using mlst (https://github.com/tseemann/mlst) and the PubMLST database [accessed on 20 March 2026] [57]. Antimicrobial resistance genes and mutation, as well as biocide resistance genes, metal resistance genes, and heat tolerance genes were identified using AMRFinderPlus v4.2.7 (database version 2026-03-24.1) [58]. Plasmid contig identification was performed using MOB-suite (v3.0.3) [59], and the location of resistance genes as plasmid or chromosome was determined by combining the AMRFinderPlus and MOB-suite results. Screening for potential virulence genes was performed using ABRicate v1.0.0 [60] and the Virulence Factors Database v2.0.1 (VFDB, 14 January 2025) [61]. Data was visualized using R v4.5.3 [50]. All WGS data from the 387 ESBL *E. coli* isolates were deposited in the Sequence Read Archive under BioProject PRJNA1450717 (https://www.ncbi.nlm.nih.gov/bioproject/1450717) [accessed on 9 May 2026]. For isolates in which Shiga toxin genes were found, subtyping of *stx* genes from raw reads was conducted using K-mer Alignment (KMA) v1.4.9 [62]. Reads were mapped against the STxOP database (version 1) using a 75% identity cutoff (github link). The conclave 1 consensus scheme was used to resolve match selection for multi-mapping reads.

### 5.4. Phylogenetic Analysis

Snippy v.4.6.0 was used to generate a core single-nucleotide polymorphism (SNP) alignment, with each genome mapped to the reference strain *E. coli* AMC909 (https://github.com/tseemann/snippy) [accessed on 20 March 2026]. The resulting core alignment was analyzed using Gubbins [63] v3.4.3 to account for recombination, and a maximum-likelihood phylogeny was inferred with IQ-TREE v2.4.0 [64], applying the best-fit substitution model identified automatically (GTR+F+I+G4). Treeviewer was used to visualize the phylogenetic tree [65]. Pairwise SNP distances were calculated using snp-dists v.0.8.2 (https://github.com/tseemann/snp-dists) [accessed on 20 March 2026].

### 5.5. Statistical Analysis

Data processing and statistical analyses were performed with R v4.5.3 [50]. The tidyverse v.2.0.0 [66] package was used for data processing, and plots were generated using ggplot2 v.3.5.1 [67], ComplexHeatmap [68], and circlize [69]. The rcompanion package v.2.5.0 [70] was used to perform statistical comparisons assessing differences in farming regimes, sample sources, and seasons using chi-square tests (*p* < 0.05) and Fisher’s exact tests (Bonferroni method). Differences in the quantity of resistance genes, metal resistance genes, virulence factors, and plasmid counts per isolate among phylogroups were assessed using the Kruskal–Wallis test. When the overall test was significant (*p* < 0.05), pairwise comparisons were conducted using Dunn’s test with Benjamini–Hochberg correction. Phylogroups with low sample sizes (<5 isolates) were excluded from the analysis. Adjusted *p*-values < 0.05 were considered statistically significant.

## Figures and Tables

**Table 1:** Occurrence of ESBL *E. coli* across sample types collected in this study, 2022–2024.

Sample Type	Total Samples	ESBL Positive (%)	Confidence Interval (95%)
Calf Feces	144	106 (73.61%)	65.62–80.60%
Milking Cow Feces	144	51 (35.42%)	27.63–43.81%
Dry Cow Feces	144	65 (45.14%)	36.84–53.64%
Manure Pit	144	69 (47.92%)	39.53–56.39%
Manured Soil	96	6 (6.25%)	2.33–13.11%
Surface Water	144	27 (18.75%)	12.73–26.10%
Wastewater Influent	54	41 (75.93%)	62.36–86.51%
Wastewater Effluent	42	14 (33.33%)	19.57–49.55%

**Table 2:** Most common Sequence Types (STs) and resistance profiles among ESBL *Escherichia coli* isolates from different sample types.

Sample Type	Top STs *	Top Resistance Profiles	No. Antimicrobial Classes
Calf Feces *n* = 107	ST3018, ST10, ST69	*aadA5*, *acrF*, *aph(3″)-Ib*, *aph(6)-Id, bla_CTX-M-55_*, *bla_EC_*, *dfrA17*, *emrD*, *floR*, *mdtM*, *sul2*, *tet(A)* *n* = 17	6
Milking Cow Feces *n* = 53	ST2524, ST58, ST38	*aadA5*, *acrF*, *aph(3″)-Ib*, *aph(6)-Id*, *bla_CTX-M-55_*, *bla_EC_*, *dfrA17*, *emrD*, *floR*, *mdtM*, *sul2, tet(A)* *n* = 10	6
Dry Cow Feces *n* = 64	ST38, ST648, ST69	*acrF*, *bla_CTX-M-15_*, *bla_EC_*, *emrD*, *mdtM*, *qnrS1* *n* = 7	2
Manure Pit *n* = 70	ST2524, ST648, ST155, ST666	*aadA5*, *acrF*, *aph(3″)-Ib*, *aph(6)-Id*, *bla_CTX-M-55_*, *bla_EC_*, *dfrA17*, *emrD*, *floR*, *mdtM*, *sul2*, *tet(A)* *n* = 11	6
Manured Soil *n* = 6	-	*aac(3)-VIa*, *aadA1*, *acrF, aph(3″)-Ib*, *aph(6)-Id*, *bla_EC_*, *bla_SHV-2A_*, *emrD*, *mdtM*, *sul1*, *tet(A)* *n* = 2	4
Surface Water *n* = 28	ST3018, ST744	*aadA5*, *acrF*, *aph(3″)-Ib*, *aph(6)-Id*, *bla_CTX-M-55_*, *bla_EC_*, *dfrA17*, *emrD*, *floR*, *mdtM*, *sul2, tet(A)* *n* = 4	6
Wastewater Influent *n* = 36	ST410, ST73, ST10, ST517	*aadA1*, *acrF*, *bla_CTX-M-15_*, *bla_EC_*, *bla_TEM_*, *emrD*, *sul1* *n* = 5	3
Wastewater Effluent *n* = 23	ST38, ST443, ST131, ST69	*acrF*, *bla_CMY-42_*, *bla_DHA-1_*, *bla_EC_*, *catA1*, *emrD*, *erm(B)*, *mdtM*, *mph(A)*, *mph(E)*, *mrx(A)*, *msr(E)*, *qnrB4*, *sul1*, *tet(B)* *n* = 3	6

* The three most common STs per sample type. Manured soil isolates included only singleton STs.

**Table 3:** Summary of the toxin genes and singleton virulence factors among ESBL *Escherichia coli* (*n* = 387) from different sample types.

Sample Type	Toxin Genes (*n* *)	No. Toxin Genes	Singleton Virulence Genes **	No. Singleton Genes
Calf Feces	*astA* (15), *cdtA* (1), *cdtB* (1), *cdtC* (1), *hlyA* (10), *senB* (3), ***stx1B*** (2), ***stx2A*** (8), ***stx2B***(8), ***stxA*** (2), *toxB* (7)	11	*cdtA*, *cdtB*, *cdtC*, *chuX*, *cnf1*, *espJ*, *espN*, *espX7*/*nleL*, *gtrB*, *nleD*	10
Milking Cow Feces	*astA* (4), *cdtA* (1), *cdtB* (1), *cdtC* (1), *estIa* (2), *hlyA* (4), *senB* (1)	7	*cdtA*, *cdtB*, *cdtC*, *cnf1*, *espF*, *espL2*, *faeC*, *faeD*, *faeF*, *faeH*, *faeI*, *faeJ*, *nleA*/*espI*, *nleB1*, *nleB2*, *nleE*, *nleH1*, *ospG*, *papB*, *papC*, *papD*, *papE*, *papH*, *papI*, *papJ*, *papK*, *senB*, *sfaX*	28
Dry Cow Feces	*astA* (7), *cdtA* (1), *cdtB* (1), *cdtC* (1)	4	*cdtA*, *cdtB*, *cdtC*, *espX6*, *hlyA*, *hlyB*, *hlyC*, *hlyD*	8
Manure Pit	*astA* (4), *estIa* (1), *hlyA* (2), *sat* (2), *toxB* (1)	5	*east1*, *efa1*, *espB*, *espJ*, *espL2*, *espM1*, *espN*, *espR3*, *espX2*, *espX7*/*nleL*, *estIa*, *faeC*, *faeF*, *faeH*, *faeI*, *faeJ*, *nleA*, *nleA*/*espI*, *nleB1*, *nleE*, *nleH2*, *sepZ*/*espZ*, *shuS*, *shuY*, *toxB*	25
Manured Soil	*astA* (2), *cdtA* (1), *cdtB* (1), *cdtC* (1)	4	*cdtA*, *cdtB*, *cdtC*, *chuY*, *cnf1*, *espR3*, *f17d-C*, *f17d-D*, *f17d-G*, *gtrA*, *gtrB*, *hlyC*, *iucA*, *iucB*, *iucC*, *iucD*, *iutA*, *shuY*	18
Surface Water	***stx1B*** (1), ***stxA*** (1)	2	*espR3*, *papB*, *papF*, *papG*, *sfaX*, *stx1B*, *stxA*, *tcpC*, *vat*	9
Wastewater Influent	*astA* (1), *sat* (4), *senB* (7)	3	*afaA*, *afaD*, *afaE-II*, *astA*, *daaF*, *draE2*, *draP*, *faeC*, *faeD*, *faeE*, *faeF*, *faeH*, *faeI*, *faeJ*, *papE*, *sfaE*, *sfaF*, *sfaG*, *sfaH*, *sfaS*, *tcpC*	21
Wastewater Effluent	*sat* (4), *senB* (3)	2	*chuA*, *chuX*, *espR3*, *espX2*, *espX6*, *f17d-A*, *f17d-C*, *f17d-D*, *f17d-G*, *papC*, *papD*, *papE*, *papG*, *papH*, *papJ*, *papK*, *shuS*, *shuY*	18

* Number of isolates possessing the specified toxin gene. ** Virulence factors found only once in our collection of isolates.

**Table 4:** Primer sequences used for PCR amplification of ESBL genes in *E. coli* isolates.

Reaction	Amplicon	Primer	Primer Sequence (5′–3′)	Amplicon Size (bp)	Reference
uspA simplex	*uspA*	Forward	CCGATACGCTGCCAATCAGT	884	[45]
		Reverse	ACGCAGACCGTAGGCCAGAT		
ESBL Multiplex 1	*blaCMY-2*	Forward	GACAGCCTCTTTCTCCACA	1000	[49]
		Reverse	TGGACACGAAGGCTACGTA		
	*blaOXA-1*	Forward	GGCACCAGATTCAACTTTCAAG	564	[47]
		Reverse	GACCCCAAGTTTCCTGTAAGTG		
	*blaSHV*	Forward	CTT TAT CGG CCC TCA CTC AA	237	[48]
		Reverse	AGG TGC TCA TCA TGG GAA AG		
	*blaTEM*	Forward	CGC CGC ATA CAC TAT TCT CAG AAT GA	445	
		Reverse	ACG CTC ACC GGC TCC AGA TTT AT		
ESBL CTX-M Multiplex 2	*blaCTX-M* group 1 variants including *CTX-M-1*, *CTX-M-3*, and *CTX-M-15*	Forward	TTAGGAARTGTGCCGCTGYA	688	[47]
		Reverse	CGATATCGTTGGTGGTRCCAT		
	*blaCTX-M* group 2 variants, including *CTX-M-2*	Forward	CGTTAACGGCACGATGAC	404	
		Reverse	CGATATCGTTGGTGGTRCCAT		
	*blaCTX-M* group 9 variants, including *CTX-M-9* and *CTX-M-14*	Forward	TCAAGCCTGCCGATCTGGT	561	
		Reverse	TGATTCTCGCCGCTGAAG		
blaCTX-M-15 simplex	*blaCTX-M-15*	Forward	ATG TGC AGY ACC AGT AAR GTK ATG GC	593	[46,48]
		Reverse	TGG GTR AAR TAR GTS ACC AGA AYC AGC GG		

## Data Availability

All Illumina sequence read data from the current study were deposited in the NCBI database as Short Read Archive (SRA) under BioProject PRJNA1450717 (https://www.ncbi.nlm.nih.gov/bioproject/1450717) [accessed on 9 May 2026].

## Supplementary Materials

The following supporting information can be downloaded at: https://www.mdpi.com/article/10.3390/antibiotics15060588/s1, Table S1. Whole Genome Sequencing Summary; Table S2. Pairwise SNP differences of ESBL *Escherichia coli* (n = 387) obtained in this study. AMC909 was used as the reference genome.
